# N^6^-methyladenosine (m^6^A) methyltransferase METTL3-mediated LINC00680 accelerates osteoarthritis through m^6^A/SIRT1 manner

**DOI:** 10.1038/s41420-022-00890-0

**Published:** 2022-05-02

**Authors:** Jiangdong Ren, Yicheng Li, Shalitanati Wuermanbieke, Shu Hu, Guangxin Huang

**Affiliations:** 1 Department of Joint Surgery, Center for Orthopaedics Surgery, The Third Affiliated Hospital of Southern Medical University (Academy of Orthopaedics Guangdong Province), Tianhe District, Guangzhou, Guangdong Province China; 2 Orthopaedics Hospital of Guangdong Province, Tianhe District, Guangzhou, Guangdong Province China; 3 Department of Orthopaedics, First Affiliated Hospital of Xinjiang Medical University, Urumqi, Xinjiang China; 4 Karamay Central Hospital, Karamay, Xinjiang China

**Keywords:** Long non-coding RNAs, Osteoarthritis

## Abstract

Increasing evidence suggest the biological roles of N^6^-methyladenosine (m^6^A) and long noncoding RNAs (lncRNAs) in the bone disease, especially osteoarthritis (OA). However, the interaction of m^6^A and lncRNA in osteoarthritis is still unclear. Here, we found that a m^6^A-related lncRNA LINC00680 upregulated in the OA tissue and IL-1β-induced isolated primary chondrocytes. Functionally, in IL-1β-induced chondrocytes, silencing of LINC00680 recovered the proliferation and repressed the extracellular matrix (ECM) degradation. Mechanistically, m^6^A methyltransferase METTL3 combined tithe the m^6^A site of LINC00680 to up-regulate its expression. Moreover, LINC00680 interacted with SIRT1 mRNA through binding at m^6^A site on SIRT1 mRNA 3′-UTR, thereby enhancing the stability of SIRT1 mRNA. Overall, these findings exhibited a role of LINC00680/m^6^A/SIRT1 mRNA complex in chondrocytes. Taken together, the present study intends to uncover the mechanism by which METTL3-mediated LINC00680 accelerates OA progression, which may provide novel insight for OA.

## Abstract (web summary)

LINC00680/m6A/IGF2BP2/SIRT1 axis promotes the OA progression in chondrocyte.

## Graphical abstract

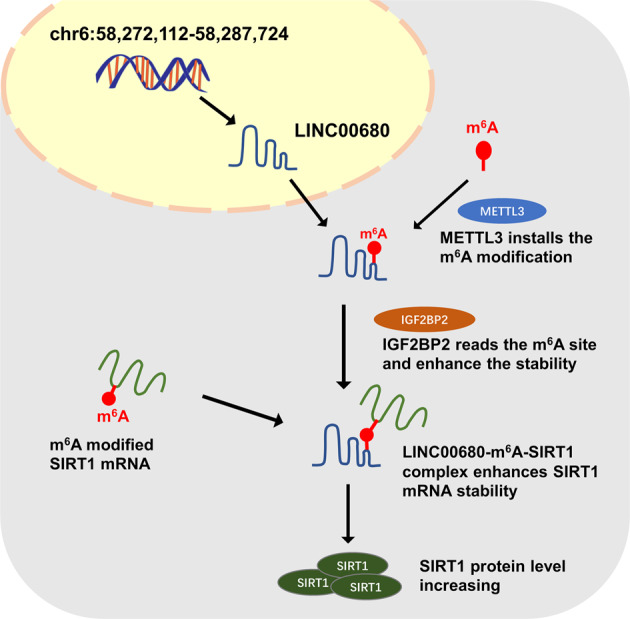

## Introduction

Osteoarthritis (OA) is chronic joints’ disease, which is characterized by progressive articular cartilage degenerative alterations and associated with biological factors and biomechanical [1, 2]. The typical clinical features of OA are degeneration of articular cartilage and secondary hyperosteogeny. Moreover, the microscopic environment changes for OA relate to chondrocytes damage and cartilage matrix degradation. Clinically, OA causes influential negative impacts on patients and reduces quality of life [3]. The apoptosis and senescence of chondrocytes trigger the extracellular matrix (ECM) degradation [4]. Importantly, the understanding for chondrocyte apoptosis mechanism and ECM degradation may be of great value for OA treatment.

Long noncoding RNAs (lncRNAs) are a group of noncoding transcripts that participate in numerous pathophysiological processes. For the joints degenerative disease, emerging evidence suggests that lncRNAs have been identified to regulate the apoptosis and extracellular matrix degradation in OA [5, 6]. For example, lncRNA MALAT1 is upregulated in IL-1β-stimulated chondrocytes in vitro and vivo and MALAT1 overexpression promotes the AKT3 expression through negatively regulating miR-150-5p [7]. LncRNA MFI2-AS1 is increased in OA tissues and LPS-induced C28/I2 cells and MFI2-AS1 knockdown attenuates LPS-induced apoptosis, viability suppression and extracellular matrix degradation [8]. LncRNA SNHG9 is downregulated in OA and SNHG9 overexpression decreases the apoptosis of chondrocytes derived from OA patients [9]. In conclusion, the evidence illuminates that lncRNAs play critical roles on OA.

N^6^-methyladenosine (m^6^A) is the most common modification occurring in eukaryotic mRNAs [10]. M^6^A modification broadly distributes in diverse pathophysiological process, which is installed by ‘writers’ refer to the RNA methyltransferases, including methyltransferase-like 3 (METTL3), methyltransferase-like 14 (METTL14), Wilms Tumor 1-associated protein (WTAP), RNA-binding motif 15 (RBM15) [11, 12]. For example, in IL-1β-treated chondrocytes, inflammatory cytokines are obviously reduced in the METTL3 overexpression group, while IL-1β administration reverses the reduced cytokines by METTL3 overexpression [13]. In IL-1β-induced ATDC5 cells, METTL3 mRNA, as well as the m^6^A methylated mRNA of total mRNA, are increased. METTL3 silencing reduces the IL-1β-induced apoptosis and inflammatory cytokines levels through activating NF-κB signaling in chondrocytes [14]. Thus, the evidence indicates that m^6^A could regulate the progression of OA.

To date, the function and mechanism by which m^6^A modulates the pathophysiological process of OA are still unclear. Here, the present research focuses on the function of m^6^A-mediated LINC00680 in the IL-1β-induced chondrocyte OA model, and investigates the potential mechanism. Present research discovered that LINC00680/m6A/SIRT1 mRNA complex regulated the proliferation of chondrocytes and ECM degradation in OA. Taken together, the present study intends to uncover the mechanism by which METTL3-mediated LINC00680 accelerates OA progression, which may provide novel insight for OA.

## Results

### LncRNA LINC00680 was induced by METTL3 in IL-1β-treated chondrocyte

**Fig. 1:**
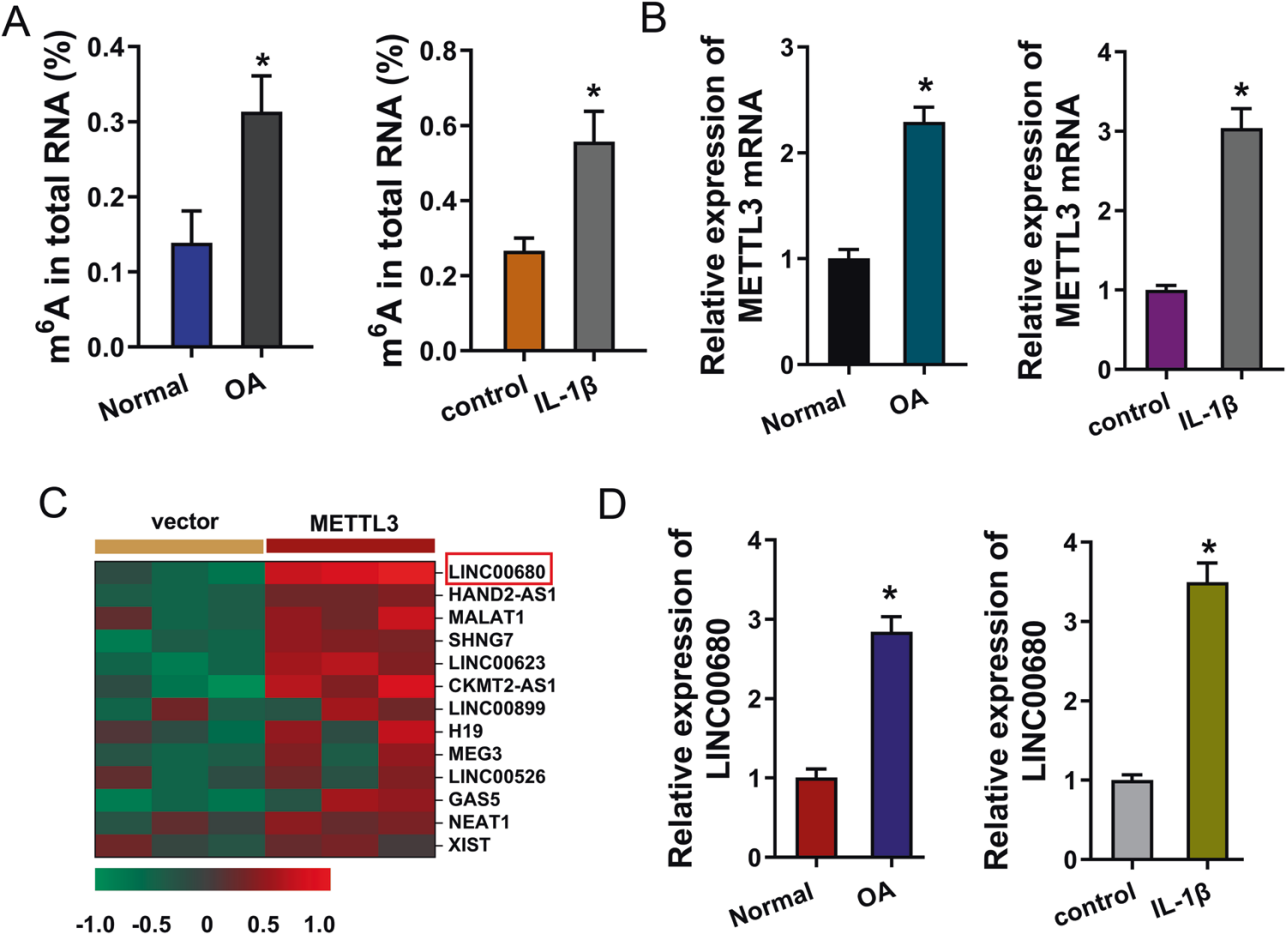
LncRNA LINC00680 was induced by METTL3 in IL-1β-treated chondrocyte. **A** m^6^A modification of RNAs was determined by m^6^A ELISA kit (Colorimetric assay). OA and normal groups respectively indicated the cartilage tissues collected from OA patients and no-OA patients. Control and IL-1β group indicated the chondrocytes treated with IL-1β (10 ng/mL) or blank. **B** METTL3 mRNA levels were detected using RT-PCR in cartilage tissues or IL-1β-treated chondrocyte. **C** Heat map showed the candidate lncRNAs’ levels detected using RT-PCR. **D** LINC00680 level was detected using RT-PCR in cartilage tissues or IL-1β-treated chondrocyte. Experiments’ data were repeated in triplicate. *Indicates the *p* < 0.05.

Clinically, the osteoarthritis (OA) cartilage tissues were collected. Besides, in order to construct the cellular model of OA, primary chondrocytes were isolated and treated by IL-1β (10 ng/mL) for 24 h. In the OA cartilage tissue and cellular model, the m^6^A modification quantitative analysis revealed that the m^6^A modification levels were increased as compared to the control group (Fig. 1A). Moreover, METTL3 acts as a critical m^6^A methyltransferase (‘writer’) and has been reported to participate in the OA pathophysiological process. Thus, our research first tried to investigate the role of METTL3 in OA. In the OA cartilage tissue and cellular model, expression levels of METTL3 were both upregulated as compared to the control group (Fig. 1B). To discover the potential downstream targets of METTL3, our research detected several candidate lncRNAs, which are reported to be related to OA, upon METTL3 overexpression transfection (Fig. 1C). Among these candidate lncRNAs, LINC00680 exhibited a remarkable overexpression than others, thus, our research selected LINC00680 for further research. In the OA cartilage tissue and cellular model, expression levels of LINC00680 were both upregulated as compared to the control group (Fig. 1D). Overall, these findings demonstrated that lncRNA LINC00680 was induced by METTL3 in IL-1β-treated chondrocyte and OA tissue.

### METTL3 enhanced the stability of LINC00680 in IL-1β-induced chondrocyte via m6A-dependent manner

**Fig. 2:**
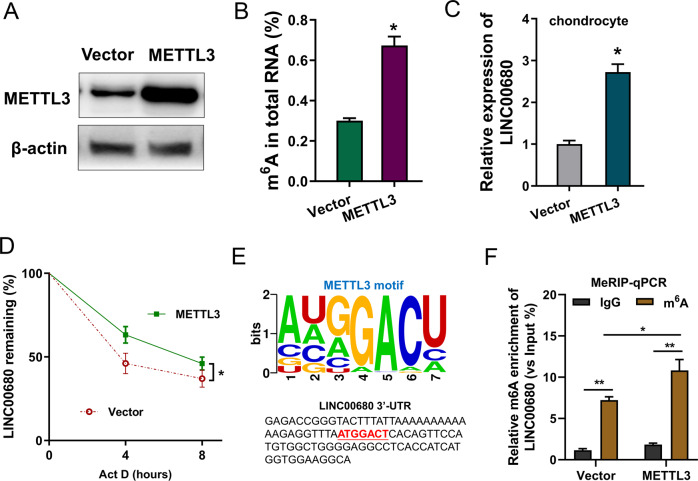
METTL3 enhanced the stability of LINC00680 in IL-1β-induced chondrocyte. **A** Western blot analysis detected the METTL3 protein level in IL-1β-induced chondrocyte transfected with METTL3 overexpression plasmids. **B** m^6^A modification quantitative analysis indicated the m^6^A modification enrichment upon the METTL3 overexpression as compared to control. **C** RT-qPCR analysis was performed to quantify the LINC00680 expression in chondrocyte upon the METTL3 overexpression as compared to control. **D** Act D treatment (2 mg/mL) for RNA stability was performed to detect LINC0068 expression using RT-qPCR. **E** Online predictive tools (RMBase, http://rna.sysu.edu.cn/rmbase/) demonstrated the m^6^A modified site in 3′-UTR of LINC00680. The m^6^A motif matched with METTL3 is GGACU. **F** MeRIP-qPCR illustrated the enrichment of m^6^A modification on LINC00680 in chondrocyte upon the METTL3 overexpression as compared to control. Experiments’ data were repeated in triplicate. *Indicates the *p* < 0.05, and **Indicates the *p* < 0.01.

In the IL-1β-induced chondrocyte (OA cellular model), the METTL3 overexpression transfection was constructed (Fig. 2A). m^6^A modification quantitative analysis revealed that the m^6^A modification levels were increased upon the METTL3 overexpression (Fig. 2B). Moreover, METTL3 overexpression accelerated the LINC0068 level (Fig. 2C). RNA stability assay demonstrated that METTL3 overexpression enhanced the stability against Act D administration (Fig. 2D). Online predictive tools (RMBase, http://rna.sysu.edu.cn/rmbase/) [15] demonstrated that there was a remarkable m^6^A modified site in the 3′-UTR of LINC00680, which was in line with the motif of METTL3 (GGACU) (Fig. 2E). MeRIP-qPCR illustrated that the m^6^A modification level of LINC00680 was increased upon METTL3 overexpression (Fig. 2F). Overall, these findings exhibited that METTL3 enhanced the stability of LINC00680 in IL-1β-induced chondrocyte via m^6^A-dependent manner.

### Knockdown of LINC00680 alleviated the proliferation repression and ECM degradation induced by IL-1β

**Fig. 3:**
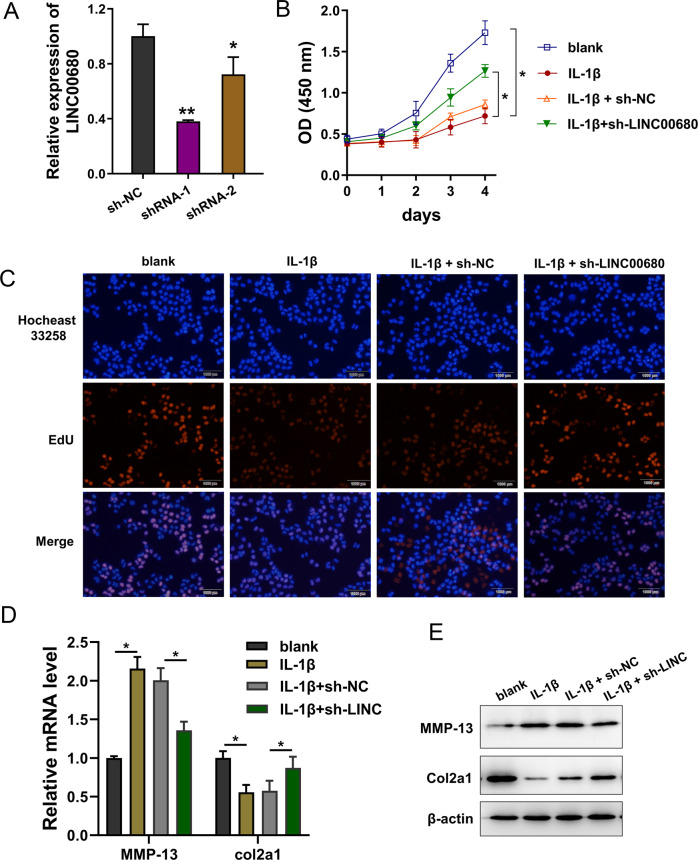
Knockdown of LINC00680 repressed the proliferation and ECM degradation. **A** LINC00680 knockdown (sh-LINC00680) was transfected into chondrocytes treated with IL-1β (10 ng/ml). RT-qPCR detected the level of LINC00680 upon sh-LINC00680 trasnfection. **B** CCK-8 proliferation assay detected the absorbance (450 nm) of chondrocytes treated with IL-1β and/or sh-LINC00680/sh-NC. **C** EdU (5′-ethynyl-2′-deoxyuridine) assay was performed for the proliferation of chondrocytes. EdU-positive cells and total Hoechst33258-positive cells were shown. **D** RT-PCR detected the mRNA levels of MMP-13 and Col2a1 in chondrocytes treated with IL-1β and/or sh-LINC00680/sh-NC. **E** Western blot analysis detected the MMP-13 protein and Col2a1 protein levels. Experiments’ data were repeated in triplicate. *Indicates the *p* < 0.05, and **Indicates the *p* < 0.01.

To investigate the function of LINC00680 on OA, the chondrocytes were treated with IL-1β (10 ng/ml) and then transfected with LINC00680 knockdown (sh-LINC00680) to construct the LINC00680 silencing (Fig. 3A). CCK-8 proliferation assay indicated that LINC00680 knockdown alleviated the proliferation repression induced by IL-1β (Fig. 3B). EdU assay indicated that LINC00680 knockdown partly recovered the proliferative ability inhibited by IL-1β (Fig. 3C). In OA, MMP-13 (catabolic factor) and type II collagen (Col2a1, anabolic factor) were considered as critical markers for ECM degradation [16–18]. The mRNA levels of MMP-13 and Col2a1 were detected using RT-PCR and results showed that LINC00680 knockdown reduced the MMP-13 mRNA level and accelerated Col2a1 level (Fig. 3D). Moreover, western blot analysis found that LINC00680 knockdown decreased the MMP-13 protein level and p-regulated the Col2a1 protein level in IL-1β administration (Fig. 3E). Overall, these findings identified that knockdown of LINC00680 alleviated the proliferation repression and ECM degradation induced by IL-1β.

### LINC00680 positively regulated the stability of SIRT1 mRNA

**Fig. 4:**
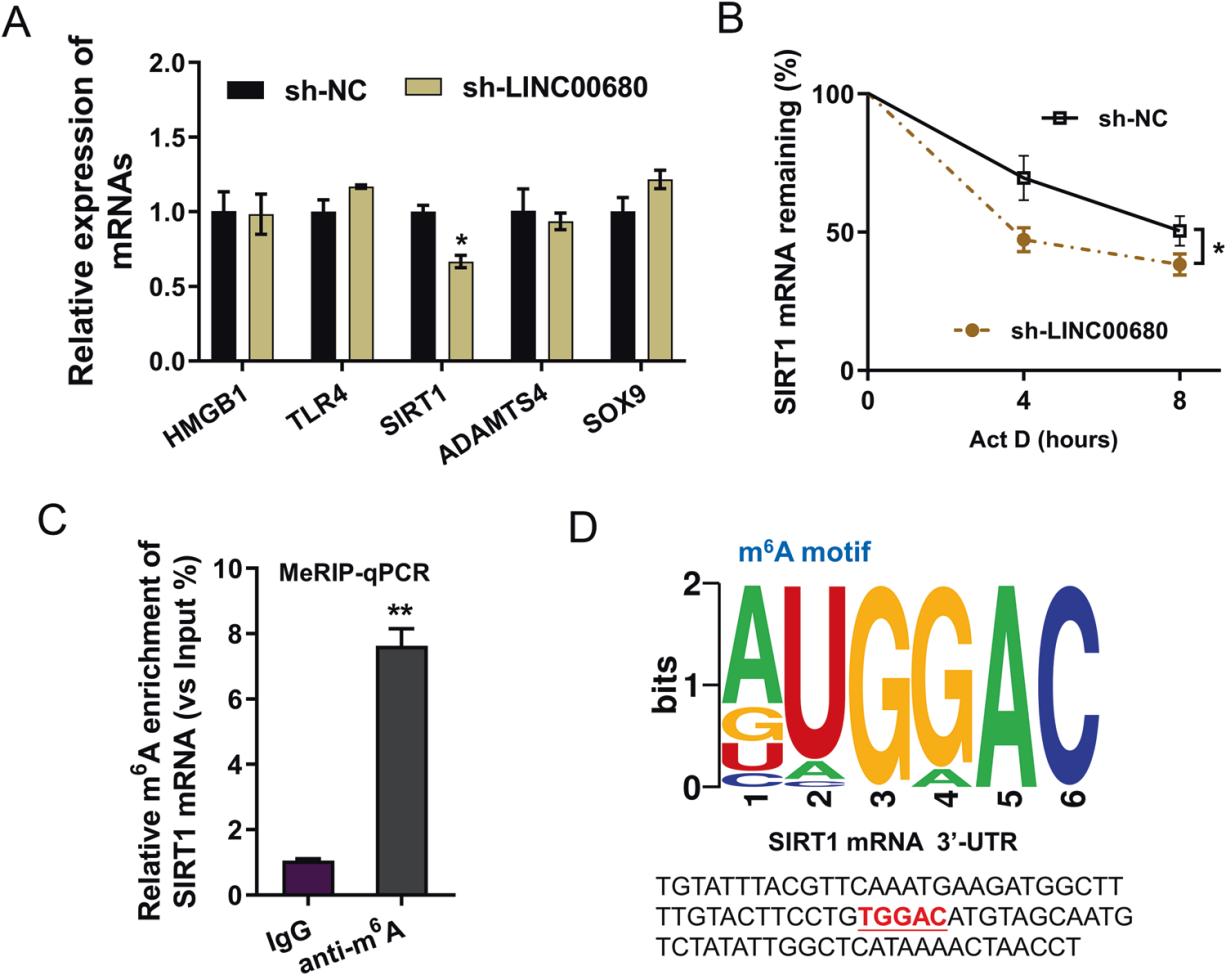
LINC00680 positively regulated the stability of SIRT1 mRNA. **A** RT-PCR detected the levels of candidate mRNAs upon LINC00680 knockdown (sh-LINC00680). **B** RNA stability analysis revealed the SIRT1 mRNA stability in chondrocytes when administrated with Act D (2 mg/mL)), including LINC00680 knockdown and control. **C** MeRIP-qPCR assay followed by qRT-PCR revealed the SIRT1 mRNA m^6^A modification. **D** Online predictive tools (RMBase, http://rna.sysu.edu.cn/rmbase/) revealed that the m^6^A motif in the 3’-UTR of SIRT1 mRNA. Experiments’ data were repeated in triplicate. *Indicates the *p* < 0.05. **Indicates the *p* < 0.01.

To investigate the potential targets of LINC00680, several candidate mRNAs were selected to detect their level in chondrocytes transfected with LINC00680 knockdown (sh-LINC00680). Results demonstrated that a candidate mRNA, SIRT1 mRNA, was remarkably decreased upon LINC00680 knockdown (Fig. 4A). RNA stability analysis found that LINC00680 knockdown reduced the SIRT1 mRNA stability when administrated with Act D (Fig. 4B). MeRIP assay followed by qRT-PCR (MeRIP-qPCR) revealed that the SIRT1 mRNA m^6^A modification level upregulated as compared to normal control (IgG) (Fig. 4C). Online predictive tools (RMBase, http://rna.sysu.edu.cn/rmbase/) revealed that there was a significant m^6^A motif in the 3′-UTR of SIRT1 mRNA (Fig. 4D). Overall, these findings exhibited that LINC00680 positively regulated the stability of SIRT1 mRNA.

### LINC00680 positively regulated SIRT1 mRNA via IGF2BP2

**Fig. 5:**
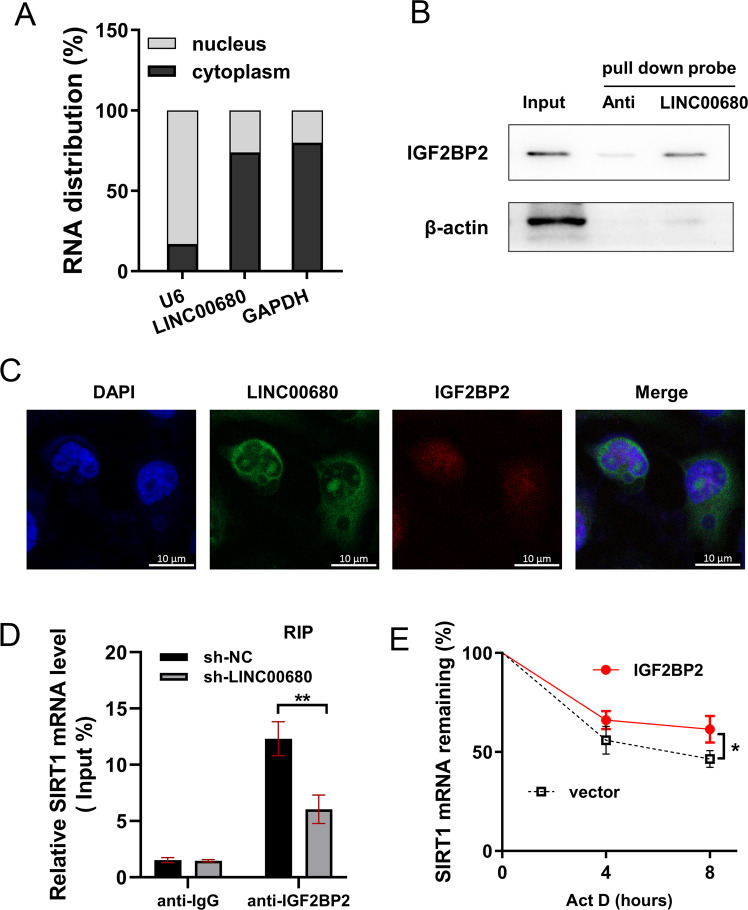
LINC00680 positively regulated SIRT1 mRNA via IGF2BP2. **A** Subcellular fractionation location was performed to detect the RNA distribution of LINC00680. **B** Biotin-labeled RNA pull down was performed in LINC00680 overexpressing transfection. Probes targeting LINC00680 and its antisense RNA were synthesized. β-actin was used as a negative control. **C** Confocal microscopy for fluorescent in situ hybridization (FISH) showed the nuclear/cytoplasm fractionation of LINC00680 and IGF2BP2 using specific probes. Nuclei were stained blue (DAPI). **D** RIP-qPCR analysis of the enrichment of SIRT1 mRNA precipitated by IGF2BP2 relative to IgG in LINC00680 knockdown or control. **E** RNA stability analysis revealed the SIRT1 mRNA stability in chondrocytes when administrated with Act D (2 mg/mL), including IGF2BP2 overexpression and control. Experiments’ data were repeated in triplicate. *Indicates the *p* < 0.05. **Indicates the *p* < 0.01.

In the chondrocytes, RNA distribution analysis found that LINC00680 mainly distributed in the cytoplasmic portion, suggesting its function of post-transcriptional regulation (Fig. 5A). Biotin-labeled RNA pull down followed western blot assay indicated that LINC00680 could interact with IGF2BP2 in chondrocytes (Fig. 5B). RNA-FISH assay found that LINC00680 and IGF2BP2 predominantly located in the cytoplasmic portion, indicating the colocalization of endogenously expressed LINC00680 and IGF2BP2 in the cytoplasm (Fig. 5C). RNA immunoprecipitation (RIP) assay indicated that LINC00680 knockdown reduced the SIRT1 mRNA level precipitated by anti-IGF2BP2, unveiling the interaction within LINC00680/IGF2BP2/SIRT1 mRNA (Fig. 5D). RNA stability analysis found that IGF2BP2 enhanced the SIRT1 mRNA stability when administrated with Act D (Fig. 5E). Overall, these findings identified that LINC00680 positively regulated SIRT1 mRNA via IGF2BP2.

## Discussion

As an indispensable type of epigenetic regulation, m^6^A modification has been found to be an essential regulator participating in the osteogenic disease. Although considerable amounts of literature have unraveled the regulation of lncRNA and m^6^A on the human pathophysiological process, their functions on OA are still unclear. Therefore, it is urgent to investigate the molecular mechanisms underlying OA progression and explore accurate targets for OA treatment.

Firstly, increasing evidence indicates that lncRNAs play critical regulation on the OA genesis. For example, in osteoarthritis patients‘ samples, lncRNA MIR4435-2HG expression increases and MIR4435-2HG overexpression promotes the proliferation of chondrocytes, while MIR4435-2HG knockdown accelerates the apoptosis of chondrocytes [19]. LncRNA DILC is downregulated in patients with osteoarthritis. LncRNA DILC overexpression inhibits IL-6 expression in chondrocytes, thereby alleviating the chondrocytes apoptosis in osteoarthritis [20]. LncRNA CASC2 level upregulates in plasma of osteoarthritis patients and CASC2 overexpression promotes IL-17 expression and inhibits the proliferation in human chondrocyte cell line (CHON-001) [21]. Taken together, the literature indicates the critical regulation of lncRNA in OA.

Emerging evidence indicates that m^6^A modification participates in the human pathophysiological process [22, 23]. As an indispensable form of epigenetic modification, m^6^A is suggested as an essential regulator regarding bone disease. Here, our research found via m^6^A quantitative analysis that the m^6^A enrichment in total RNA was higher when administrated with IL-1β. Given that METTL3 acts as a critical m^6^A ‘writer’ being responsible for installing the methylated site on adenylate (RRACH motif), we further ask whether potential lncRNAs could be regulated by METTL3 in OA. Thus, we constructed the METTL3 overexpression in chondrocytes and then detected the expression variation upon METTL3 overexpression. Results unveiled that LINC00680 level was upregulated when METTL3 was overexpressed. Further results indicated that METTL3 targeted the GGACU site, which was methylated by METTL3, on the 3′-UTR to enhance the stability of LINC00680.

Lately, studies have found that m^6^A modification could regulate the metabolism of lncRNAs [24, 25]. For example, in non-small-cell lung cancer, MeRIP-Seq discovers that there was a m^6^A modification site in ABHD11-AS1 and METTL3 promotes the ABHD11-AS1 transcript stability to increase its expression [26]. In hepatocellular carcinoma, METTL3 mediates the m^6^A modification of LINC00958, leading to the upregulation of LINC00958 through stabilizing its RNA transcript, then upregulates hepatoma-derived growth factor (HDGF) expression [27]. Moreover, specific m^6^A readers YTHDF1 and YTHDF2 read the m^6^A motifs of the lncRNA THOR and regulates its stability through m^6^A-dependent RNA–protein interactions [28]. Collectively, these findings highlight the critical role of the m6A modified lncRNA in human pathophysiological process.

In the pathogenesis of OA, Silent Information Regulator 2 type 1 (SIRT1) has been identified to related to age-associated diseases, such as diabetes type II, Alzheimers and osteoporosis [29]. Given the definite functions of SIRT1 in OA, our study laid more emphasis on the manner by which LINC00680 regulated SIRT1 in OA. Here, our results demonstrated that METTLL3-induced LINC00680 could bind with the SIRT1 mRNA through the interaction of IGF2BP2 at m^6^A modification site.

In conclusion, our study demonstrates that m^6^A-mediated LINC00680 regulates the proliferation and ECM degradation of chondrocytes through LINC00680/m6A/SIRT1 mRNA axis. Taken together, present study intends to uncover the mechanism by which METTL3-mediated LINC00680 accelerates OA progression, which may provide novel understanding of the role of m^6^A and lncRNA in OA.

## Materials and methods

### Cartilage specimens

OA cartilage tissues were obtained from OA patients who had undergone total knee replacement. Control normal cartilage tissues were obtained from patients underwent total hip replacement surgery with femoral neck fractures. The study had been approved by the Ethics Committee of First Affiliated Hospital of Xinjiang Medical University. Written informed consent was obtained from all patients.

### Chondrocytes and culture

Chondrocytes were isolated from human cartilage and cultured according to a previous study [30]. In brief, after centrifugation, chondrocytes were isolated from the pieces of digested cartilage tissue samples. Then, isolated chondrocytes were cultured in DMEM-F12 supplemented 10% fetal bovine serum (FBS, Gibco) and antibiotics.

### Transfection

For the silencing of LINC00680, lentivirus vectors containing LINC00680 specific shRNA were provided by OBiOc (Shanghai, China). In brief, shRNA sequences targeting human METTL3 were cloned into a pLKD-CMV-G&PR-U6-shRNA vector. Chondrocytes were infected with the lentivirus with Polybrene (5 mg/mL), and then selected by puromycin (2 mg/mL) in culture medium. For the overexpression of METTL3 and IGF2BP2, the whole sequences of them were synthesized and sub-cloned into a pcDNA3.1 (+) vector (GenePharma, Shanghai, China). The transfection efficiency was evaluated with qRT-PCR. The shRNA sequences were listed in Table S1.

### m^6^A quantification

Total RNA was isolated from chondrocytes using TRIzol (Invitrogen) according to the manufacturer’s instructions. The quality of RNA was analyzed by NanoDrop (). The quantification of m^6^A in total RNA was identified using m^6^A RNA methylation quantification kit (ab185912; Abcam) according to the manufacturer’s instructions. In brief, the RNAs (200 ng) were coated by capture antibody solution in a suitable diluted concentration following the manufacturer’s instructions. The m^6^A level was colorimetrically quantified according to the absorbance at a wavelength of 450 nm.

### Western blot

Total protein was extracted from chondrocytes using radio-immunoprecipitation assay (RIPA) lysis buffer containing protease inhibitor cocktail. Protein concentration was determined using bicinchoninic acid (Thermo Fisher) assay. Protein lysate (30 μg) was resolved using 10% sodium dodecyl sulphatepolyacrylamide gel electrophoresis (SDS-PAGE) and then transferred to PVDF (polyvinylidene difluoride) membranes. Then, membrane was blocked with 5% nonfat milk with TBST (0.05 M Tris, 0.15 M NaCl, pH 7.5) and Tween-20 (0.2%) for 1 h. Membrane was incubated with primary antibodies (anti-METTL3, #86132, CST, 1:1000; anti-Collagen II, ab188570, Abcam, 1:1000; anti-MMP13, Abcam, ab51072, 1:1000; anti-IGF2BP2, CST, #14672, 1:1000) diluted in TBST overnight at 4 °C. After incubation by secondary antibodies, the members were detected by Odyssey infrared imaging system (LI-COR, Inc., Lincoln, NE, USA) and the band intensity was identified by ImageJ software.

### Ethynyl-2-deoxyuridine (EdU) incorporation assay

For the EdU incorporation assay, cells were cultured in 24-well plates and EdU (10 mM) was added to each well for an additional 2 h culture. Then, chondrocytes were fixed with 4% formaldehyde for 30 min. Once being washed, chondrocytes were incubated with glycine (50 μl, 2 mg/mL) for 5 min and washed with PBS. After permeabilization, chondrocytes were reacted with 1X Apollo solution at room temperature for 30 min in the dark. After that, chondrocytes were incubated with 100 μl of 1X Hoechst 33258 solution at room temperature for 30 min in the dark. After washing, cells were visualized under a fluorescence microscopy. The EdU incorporation rate was calculated as the ratio of EdU-positive to total DAPI-positive cells.

### CCK-8 proliferation assay

Proliferation of chondrocytes was assessed using CCK8 assay (CK04, Dojindo Laboratories, Kumamoto, Japan). Treated cells (3 × 10^3^ cells/well) were seeded into 96-well plate and then cultured for absorbance measuring at 1, 2, 3, 4 days. The absorbance was detected at 450 nm with microplate reader.

### Act D treatment for RNA stability

Treated chondrocytes were exposed to actinomycin D (2 mg/mL, Merck, Darmstadt, Germany) at indicated time (0 h, 4 h, 8 h). After cells were harvested, total RNA was extracted. After treatment, RNA was transcribed into cDNA, and the expression levels of LINC00680 and SIRT1 mRNA were determined by qRT-PCR.

### Subcellular fractionation location

Nuclear and cytosolic fractions of chondrocytes were isolated using PARIS Kit (Life Technologies, CA, USA) according to the manufacturer’s instructions. GAPDH acted as cytoplasm control, and U6 acted as nuclear control. The expression of LINC00680 was identified using RT-qPCR.

### Fluorescence in situ hybridization (FISH)

FISH assay was performed to detect the expression level and localization of LINC00680 in chondrocytes. In brief, FAM-labeled probe sequences for LINC00680, cy3-labeled probe sequences for IGF2BP2 and DAPI-labeled U6 probe were synthesized from Genepharma (Shanghai, China). FISH assay was performed using fluorescent in situ hybridization kit according to the manufacturer’s protocol (Genepharma). Nuclei were counterstained with 40,6-diamidino-2-phenylindole (DAPI). The image was got by confocal microscope (Zeiss).

### m^6^A methylated RNA immunoprecipitation PCR (MeRIP-qPCR)

To quantify the m^6^A-modified RNAs (LINC00680, SIRT1 mRNA) levels, MeRIP-qPCR was performed in chondrocytes. In brief, total RNA was isolated from chondrocytes cells. Then, protein A/G magnetic beads were conjugated with anti-IgG (Cell Signaling Technology) or anti-m^6^A antibody (Millipore, ABE572) in IP buffer (1% NP-40, 20 mM Tris pH 7.5, 140 mM NaCl, 2 mM EDTA) supplemented with RNase inhibitor and protease inhibitor for overnight at 4 °C. Total RNA (100 μg) was then incubated with the antibody in IP buffer. After precipitation, RNA was eluted from the beads with elution buffer, and then purified for further qRT-PCR assay. Fold enrichment was calculated by calculating the 2^−ΔΔCt^ to the input sample.

### RNA immunoprecipitation (RIP)

The interaction within SIRT1 mRNA and IGF2BP2 was identified using RIP. In brief, approximate 5 × 10^6^ chondrocytes with stable LINC00680 knockdown or control cells were lysed with RIP lysis buffer (Millipore) and then incubated with anti-IGF2BP2 (#14672, 1:1000, Cell Signaling Technology) or non-immunized rabbit IgG at 4 °C overnight. After incubation, the RNA-protein immunocomplexes were precipitated by protein A/G magnetic beads. With RNA purification, the purified RNA was validated by qRT-PCR.

### Statistical analysis

Data were analyzed using the SPSS 19.0 software (SPSS, Chicago, IL, USA) and GraphPad Prism 8.0 (GraphPad Software, La Jolla, CA, USA). Experiments’ data were repeated in triplicate. Student’s *t* test or two-way ANOVA was used to analyze the statistical significance of differences within groups. *P < 0.05 was considered to be statistically significant difference.

## Supplementary information

Dataset 1

Dataset 2

Dataset 3

Dataset 4

Dataset 5

Dataset 6

Dataset 7

Dataset 8

Dataset 9

Table S1
