# Supplementary figures and images for: N6-methyladenosine (m6A) methyltransferase METTL3-mediated LINC00680 accelerates osteoarthritis through m6A/SIRT1 manner
Source: Cell Death Discov. 2022 May 2;8:240. doi: 10.1038/s41420-022-00890-0 (PMC9061755; doi:10.1038/s41420-022-00890-0)

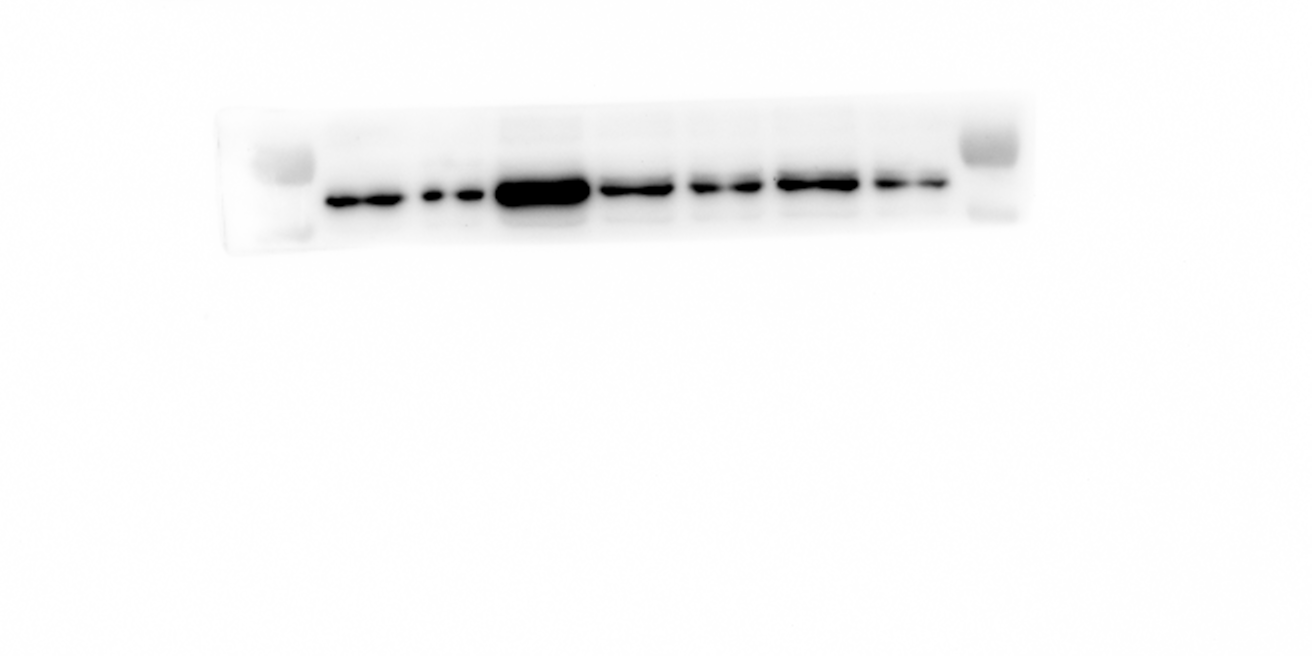

Supplement: Supplementary file 1 — Dataset 1 [file 41420_2022_890_MOESM1_ESM.tif]

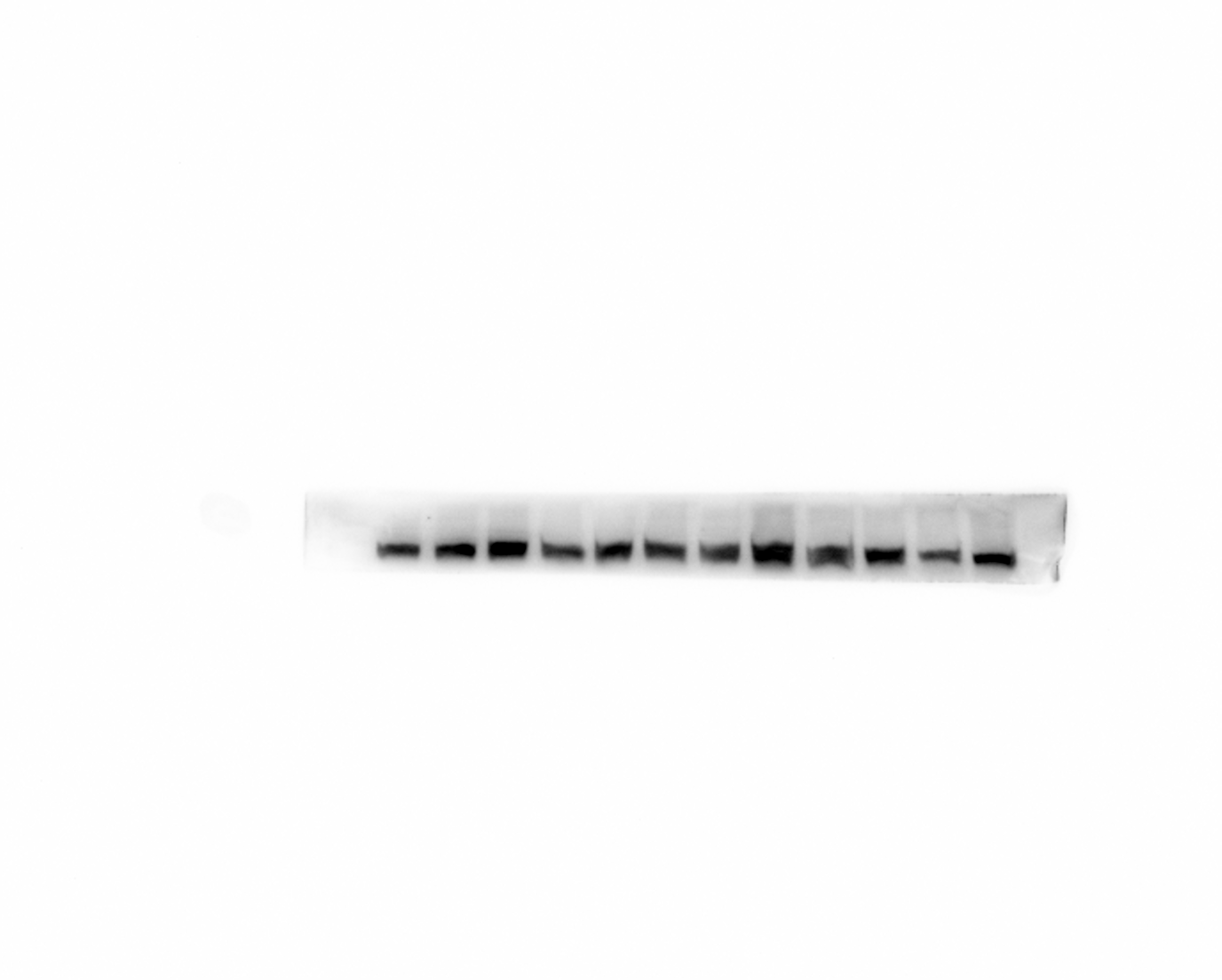

Supplement: Supplementary file 2 — Dataset 2 [file 41420_2022_890_MOESM2_ESM.tif]

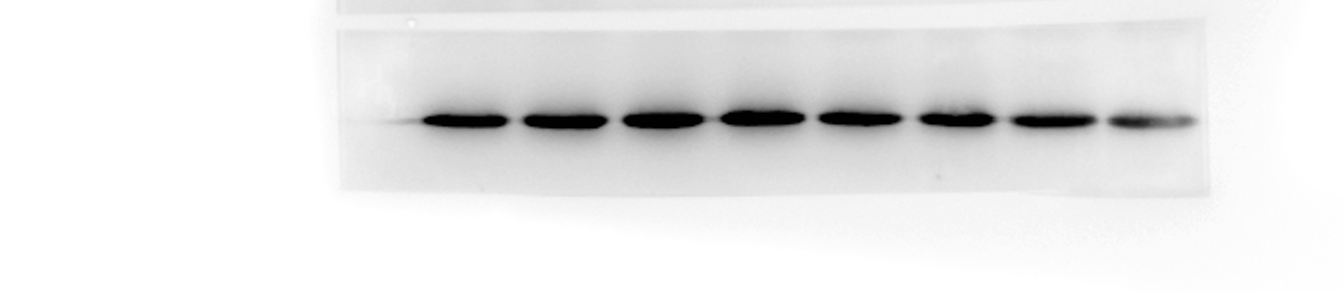

Supplement: Supplementary file 3 — Dataset 3 [file 41420_2022_890_MOESM3_ESM.tif]

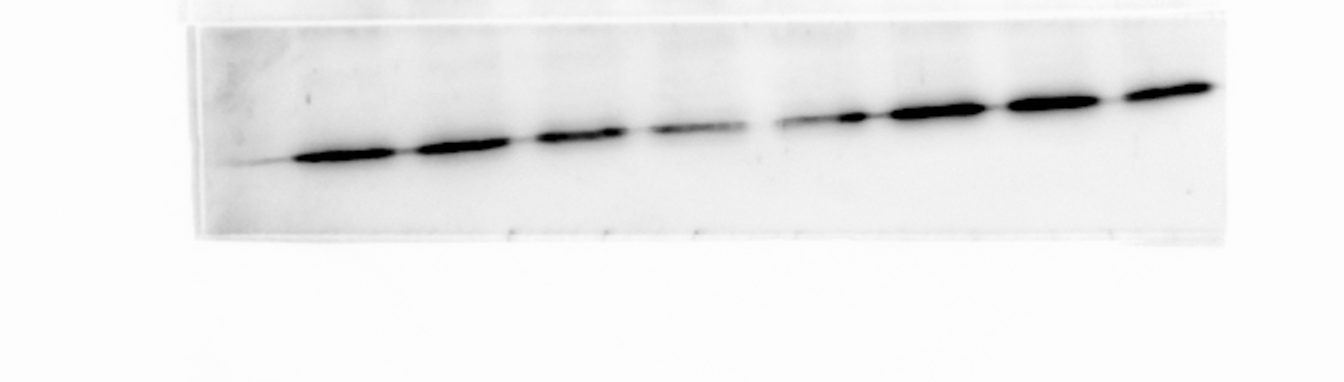

Supplement: Supplementary file 4 — Dataset 4 [file 41420_2022_890_MOESM4_ESM.tif]

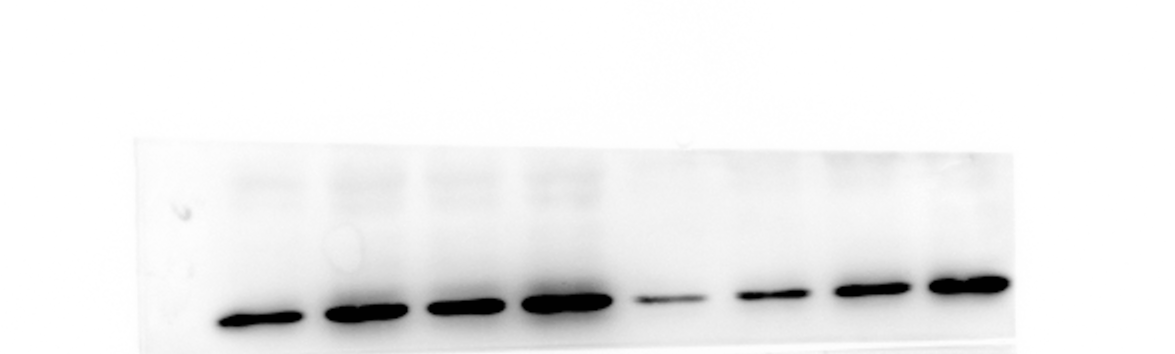

Supplement: Supplementary file 5 — Dataset 5 [file 41420_2022_890_MOESM5_ESM.tif]

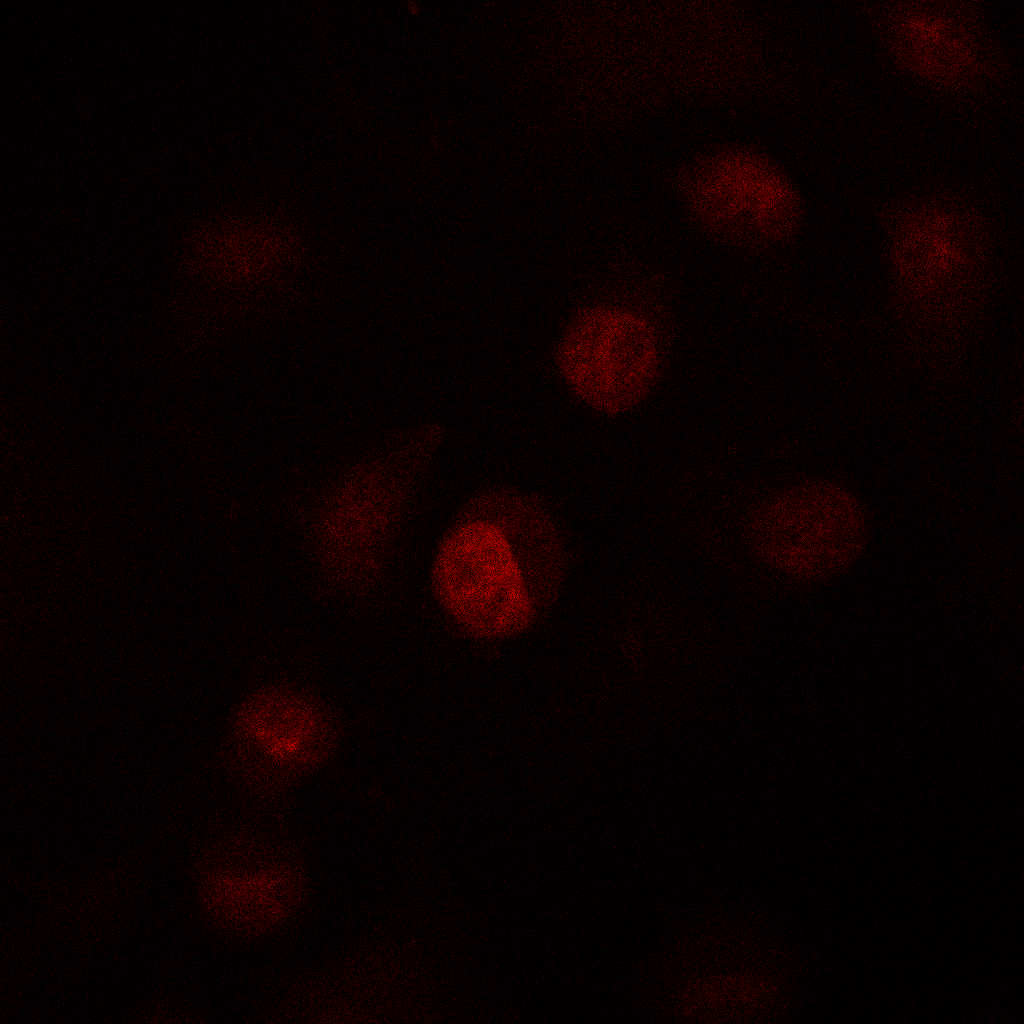

Supplement: Supplementary file 6 — Dataset 6 [file 41420_2022_890_MOESM6_ESM.tif]

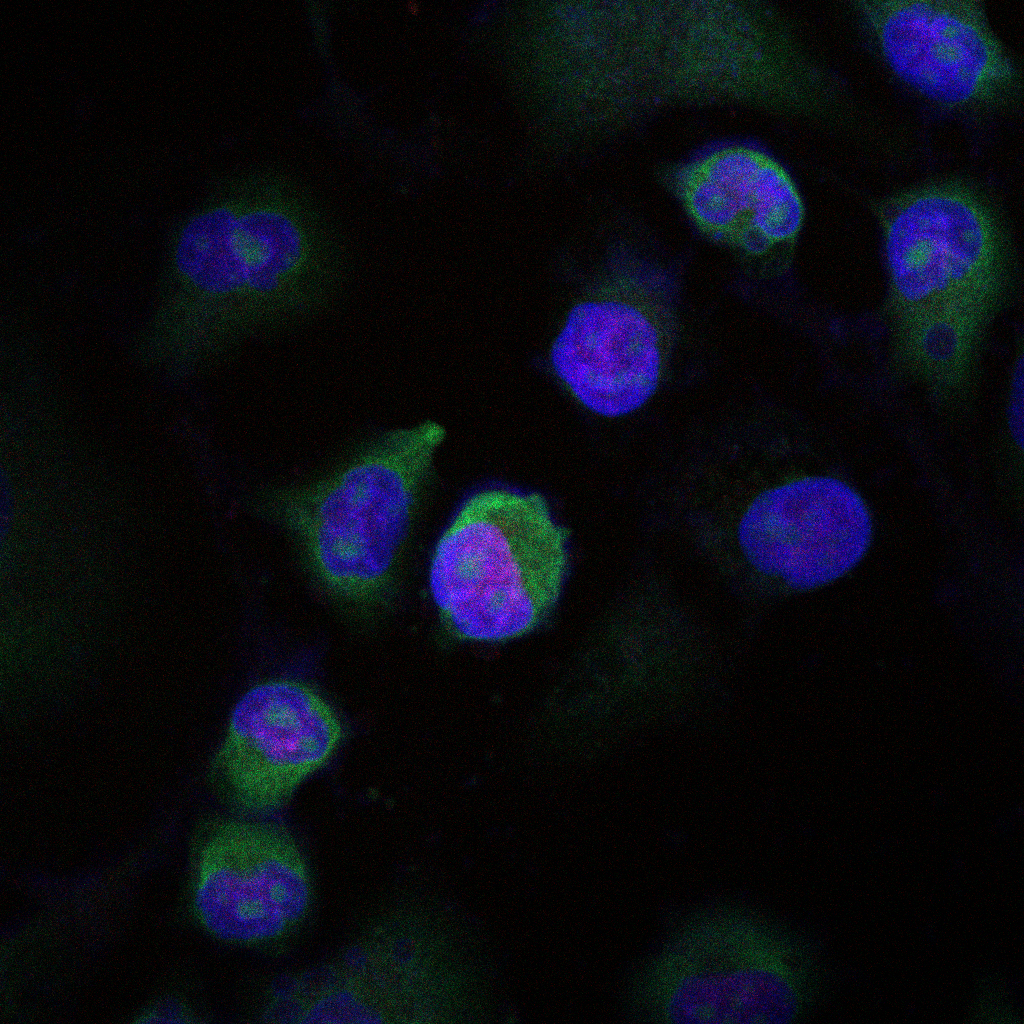

Supplement: Supplementary file 7 — Dataset 7 [file 41420_2022_890_MOESM7_ESM.tif]

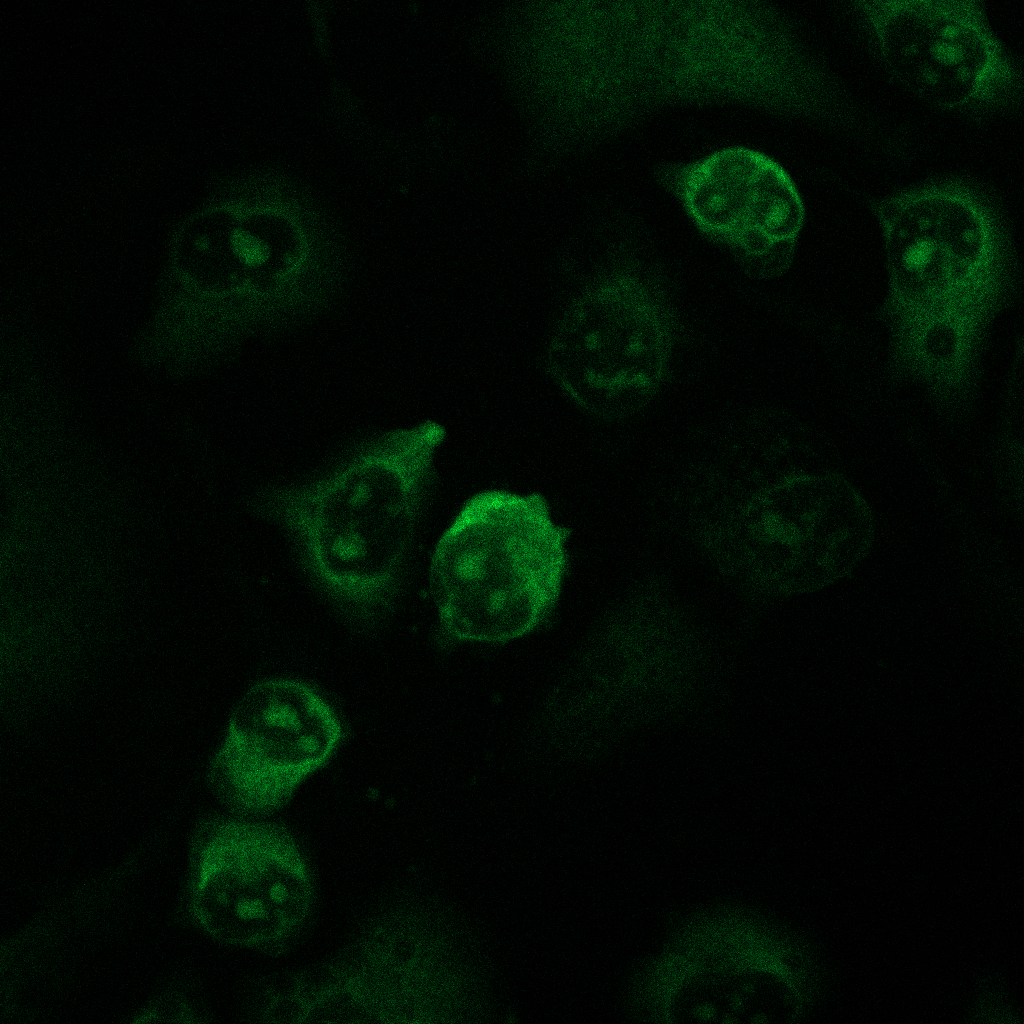

Supplement: Supplementary file 8 — Dataset 8 [file 41420_2022_890_MOESM8_ESM.tif]

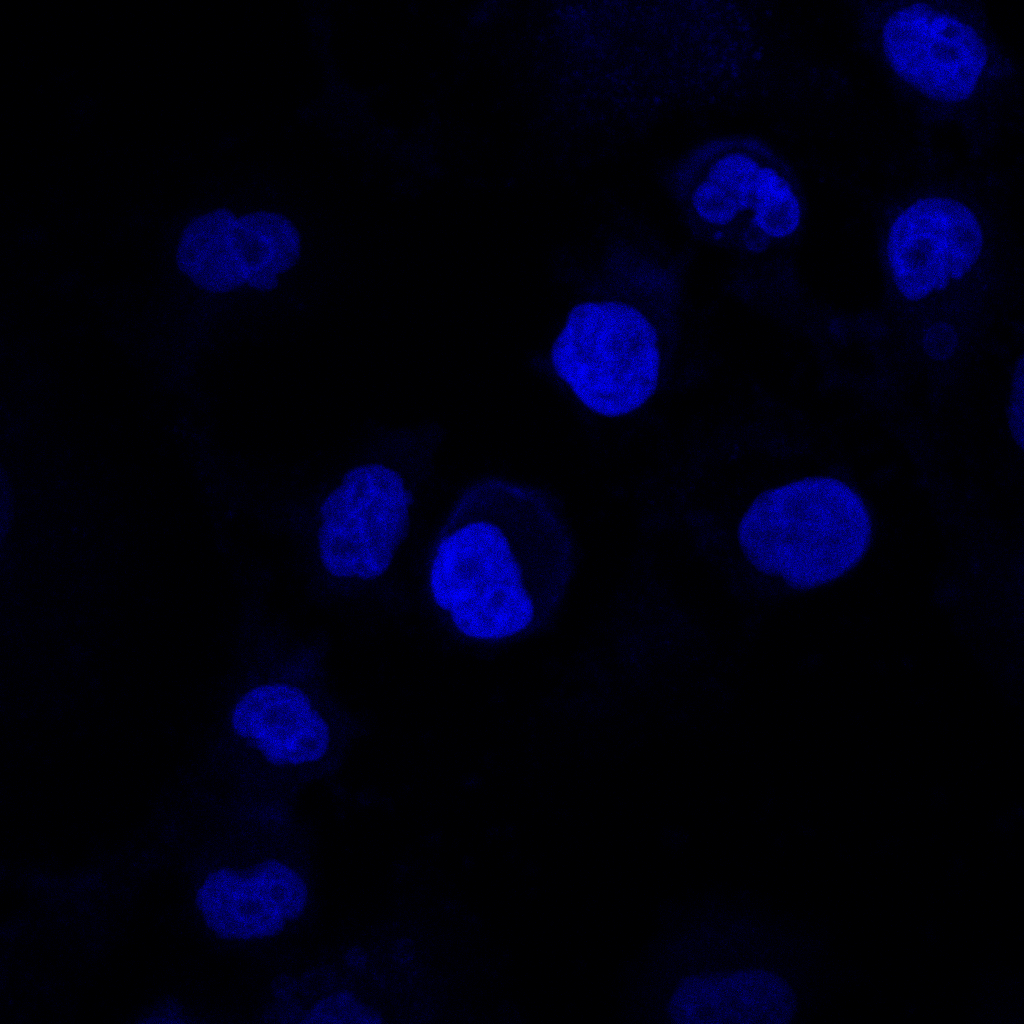

Supplement: Supplementary file 9 — Dataset 9 [file 41420_2022_890_MOESM9_ESM.tif]
