# Supplementary material for: N6-methyladenosine (m6A) methyltransferase METTL3-mediated LINC00680 accelerates osteoarthritis through m6A/SIRT1 manner
Source: Cell Death Discov. 2022 May 2;8:240. doi: 10.1038/s41420-022-00890-0 (PMC9061755; doi:10.1038/s41420-022-00890-0)
Supplement: Supplementary file 10 — Table S1 [file 41420_2022_890_MOESM10_ESM.docx]

**supplement Table S1**. Primers sequences for qRT-PCR and sequences of shRNA.

|  | Sequences |
| --- | --- |
| LINC00680 | forward, 5’-CTAGGCCGCCTCTCTTTTCC-3’  reverse, 5’-GTGTGAACACAACCCAAGGC-3’ |
| IGF2BP2 | forward, 5’-AGTGGAATTGCATGGGAAAATCA-3’  reverse, 5’-CAACGGCGGTTTCTGTGTC-3’ |
| SIRT1 | forward, 5’-TAGCCTTGTCAGATAAGGAAGGA-3’  reverse, 5’-ACAGCTTCACAGTCAACTTTGT-3’ |
| sh-LINC00680-1 | 5’- AAUAACUCGCGGCAAAUGC  UGGCAUUUGCCGCGAGUUAUUGG-3’ |
| sh-LINC00680-2 | 5’-AAGAAUAUUGACAAACUGCAA  GCAGUUUGUCAAUAUUCUUCU-3’ |
| LINC00680 probe | 5’-AAAAAACACTGCGAAGACTGTAGACACACCT  GACATCTGTGACTCTCCCAGATTCTGCGG-3’ |
| IGF2BP2 probe | 5’-AACTGCGAGGCCCTAGACCGTAACGACAT  GAAGTGACTCTCCGGTTCTCTGTAAG-3’ |
| beta-actin | forward, 5’-CTCCATCCTGGCCTCGCTGT-3’  reverse, 5’-GCTGTCACCTTCACCGTTCC-3’ |
